# Oestrogen modulators as augmentation to antipsychotics for the treatment of post- and perimenopausal psychosis: a systematic review

**DOI:** 10.1177/20451253261460148

**Published:** 2026-07-29

**Authors:** Cassidy Keen, Athanasios Hassoulas, Jill Richardson

**Affiliations:** Brunel University London, School of Medicine, Kingston Lane, Uxbridge, UK; School of Medicine, Cardiff University, Cardiff, UK; Cardiff University, School of Medicine, Cardiff, UK; School of Medicine, Cardiff University, Cardiff, UK

**Keywords:** antipsychotics, augmentation therapy, menopause, oestrogen, psychosis, raloxifene, schizophrenia, selective oestrogen receptor modulators, systematic review

## Abstract

**Background:**

It is theorised that oestrogen provides neuroprotection against psychosis. Oestrogen levels drop during post- and perimenopausal periods, creating a window for the onset of psychotic symptoms. Oestrogen treatment using hormonal therapy (HT) or selective oestrogen receptor modulators (SERMs) as an adjunct to antipsychotics could provide a new treatment option for post- and perimenopausal women.

**Objective:**

This review investigated the effectiveness of oestrogen agents as adjunctive treatment to antipsychotic medications in post- and perimenopausal women with psychosis.

**Design:**

Systematic review.

**Data sources and methods:**

A systematic search of major databases identified trials examining oestrogen or SERMs as adjuncts to antipsychotics in women aged 40+ with psychosis. Studies were assessed for quality using the Cochrane Risk of Bias tool and the Joanna Briggs Institute (JBI) critical appraisal tools.

**Results:**

Ten studies met inclusion criteria: one using oestrogen HT and nine using raloxifene. Reductions in total Positive and Negative Syndrome Scale (PANSS) scores support evidence for the adjunctive use of raloxifene in improving psychotic symptoms. Of the nine included clinical trials, six reported statistically significant improvements with oestrogen-modulating agents: four in total PANSS reduction, and two in positive PANSS domain only. Quality assessments identified publication bias, inconsistent data reporting and variability in study design.

**Conclusion:**

Raloxifene shows potential as an adjunctive treatment in post- and perimenopausal psychosis, but further high-quality clinical trials are needed to inform practice and develop hormone-informed treatment protocols.

**Trial registration:**

Not registered.

## Plain language summary

Can oestrogen-based treatments improve psychosis in perimenopausal and postmenopausal women?

Psychotic disorders such as schizophrenia can cause hallucinations, delusions, and difficulties with thought and motivation. For women, the risk of psychotic symptoms often increases around menopause, when natural oestrogen levels decline. Because oestrogen influences brain systems involved in psychosis, researchers have investigated whether oestrogen-based treatments could strengthen the effects of standard antipsychotic medication. We reviewed 10 clinical studies involving over 500 post- and perimenopausal women who received either oestrogen therapy or selective oestrogen receptor modulators (SERMs), such as raloxifene, alongside their usual treatment. Most trials focused on raloxifene. Overall, raloxifene showed modest benefits, particularly in reducing negative symptoms such as social withdrawal and lack of motivation. Results were not consistent across studies and sample sizes were small. Evidence for standard oestrogen therapy was less clear. Raloxifene was generally well tolerated in the included studies, although long term safety data is limited. These findings suggest raloxifene may be a useful adjunctive treatment for women with psychosis after menopause, but larger and more rigorous studies are needed before it can be recommended in routine practice.

## Introduction

Despite years of research, schizophrenia remains a disorder with unacceptably high rates of remission failure, desperately in need of more effective treatment modalities. The clinical picture becomes further complicated during post- and perimenopausal periods when women are at significant risk for psychiatric decompensation.^1^ From a physiological perspective, it has been theorised that these sex-dichotomous features are because of oestrogen playing a neuroprotective role.^2^ Since oestrogen increases the susceptibility threshold for developing a psychotic illness, women are at higher risk of psychiatric decompensation at stages of their lives where oestrogen levels decrease.^3^ This is displayed in postpartum psychosis and late-onset psychosis during post- and perimenopausal periods.^1^ In light of this, there is growing research into the therapeutic utility of using oestrogen as a treatment for psychosis, which has shown promising outcomes.^4^

Schizophrenia and related primary psychotic disorders are characterised by substantial impairments in perceiving reality, including delusions, hallucinations, and disorganised thinking.^5^ Symptom presentation and treatment responses differ by sex, with females generally exhibiting more positive symptoms and fewer negative symptoms than males.^6^ Women more frequently present with affective features, such as depression and anxiety, while being less responsive to antipsychotic medication during later stages in life.^6,7^ Women are also statistically more likely to have experienced childhood sexual abuse,^8^ which is associated with a 2.6-times higher likelihood of developing schizophrenia in adulthood.^9^ While onset typically occurs in late adolescence or early adulthood, women show a second peak of incidence during the menopausal transition, often between ages 40 and 50.^10^ These late-onset presentations account for up to 20% of diagnosis of schizophrenia.^11^ Consequently, the menopausal transition represents a critical window during which psychotic symptoms may emerge or worsen. Together, these intersecting biological and psychosocial vulnerabilities underscore the necessity of sex- and hormone-informed treatment approaches. However, current clinical guidelines largely fail to account for these differences,^1^ perpetuating a one-size-fits-all model of care that inadequately serves a substantial proportion of patients.

Oestrogens modulate dopaminergic, serotonergic, and glutamatergic systems implicated in the pathophysiology of psychosis. Two main theories underpin the action of oestrogen in psychotic disorders: the neuroprotection hypothesis^12^ and the hypoestrogenism hypothesis.^10,13^ The neuroprotective hypothesis proposes that oestrogen supports brain function and reduces psychosis risk. Oestrogen exerts protective effects primarily through activation of oestrogen receptor alpha (ERα) and oestrogen receptor beta (ERβ),^14^ regulating the expression and functional activity of dopamine receptors and transporters via genomic mechanisms involving receptor-mediated gene transcription and rapid intracellular signalling pathways.^15^ Oestrogen influences gene expression related to neurotransmission, glucose metabolism, and neurotrophin synthesis. Specifically, it modulates brain-derived neurotrophic factor, which plays a critical role in neuronal development, survival, and glutamatergic signalling.^16^ Dysregulation of glutamate neurotransmission, particularly excessive excitatory signalling secondary to N-methyl-D-aspartate (NMDA) receptor dysfunction, has been strongly implicated in the pathophysiology of psychosis and may represent a key target of oestrogen’s modulatory effects.^17^ Oestrogens also enhance synaptic plasticity while exerting anti-inflammatory and antioxidant effects through modulation of microglial activity and mitochondrial defence.^18^ In contrast, the hypoestrogenism hypothesis is based on early clinical observations linking schizophrenia onset and exacerbation to reproductive events and ovarian function.^13^ While early interpretations implied a direct causal role, subsequent evidence suggests that reduced oestrogen availability contributes to illness vulnerability and symptom expression rather than acting as a primary cause.^19^ Contemporary interpretations hypothesise hypoestrogenism as a downstream or contributory factor.^20^ This potentially arises from hypothalamic–pituitary–gonadal axis dysfunction or antipsychotic-induced hyperprolactinaemia, which suppresses gonadal hormone production^21^ and may attenuate oestrogen’s neuroprotective effects over time.

Hormonal augmentation strategies show promise as adjunct treatments for women experiencing psychosis during menopause. However, widespread adoption has been hindered by concerns following a study conducted by the Women’s Health Initiative (WHI, 2003),^22,23^ which linked hormone replacement therapy (HRT) to increased risks of cardiovascular disease and breast cancer. These findings, though contested, have fuelled public and clinical hesitation. As a result, selective oestrogen receptor modulators (SERMs) such as raloxifene have gained attention. SERMs act as oestrogen agonists in the brain and antagonists in breast and uterine tissue, offering a potentially safer alternative.^24,25^ Though in animals, the amounts of raloxifene entering the brain appear relatively small, in vivo assays have confirmed that these concentrations are sufficient to produce pharmacological effects.^25^ Still, its pharmacokinetics in humans remains insufficiently characterised. Alongside oestradiol-based HT and SERMs, other hormone-related interventions have been explored, including neurosteroids such as dehydroepiandrosterone (DHEA)^26^ and synthetic steroids such as Tibolone. Though there are no clinical trials to date investigating its role in psychosis, Tibolone has been shown to significantly improve depressive symptoms in perimenopausal women^27^ which supports meaningful neuropsychiatric effects.

Previous reviews investigating oestrogen augmentation in schizophrenia have primarily focused on raloxifene, often excluding oestradiol-based hormone therapies and perimenopausal populations. No synthesis to date has examined trial quality, hormonal context, pharmacogenetic moderators, and outcomes beyond symptom severity. Moreover, prior reviews have not stratified findings based on reproductive phase or investigated cognitive and genetic outcomes in depth. These gaps hinder translation into clinical practice and overlook key biological variables affecting treatment response.

This review, therefore, evaluates the efficacy of oestrogen-modulating agents as adjuncts to antipsychotic medication for the treatment of psychosis in post- and perimenopausal women. This will be done by conducting a multifactorial examination of all current registered clinical trials that use either SERMs or oestrogen HRT in the treatment of psychosis in post- and perimenopausal women. Safety and tolerability are considered secondary outcomes, alongside biological moderators of treatment response, with the secondary aim of informing recommendations for the potential use of adjunctive oestrogen treatment in standardised clinical practice

## Methods

### Study design

This study is a systematic review conducted in accordance with the Preferred Reporting Items for Systematic Reviews and Meta-Analyses (PRISMA) 2020 guidelines.^28^ PRISMA is an evidence-based tool for reporting systematic reviews and meta-analyses consisting of a 27-item checklist and a four-phase flow diagram.^28^

### Search strategy

Study screening and data extraction were conducted by a single reviewer. Although the primary systematic search was conducted in August 2022, a targeted search for newly published or registered clinical trials was performed in 2025, and no additional eligible studies were identified.

The Population, Intervention, Comparison, Outcomes and Study (PICOS) framework model was used to develop the research question and target population to facilitate the literature search (Table 1). PICOS is a structural tool that frames clinical questions according to the five types of clinical information- population, intervention, comparison, outcomes and study design.^29^ This review was not prospectively registered.

**Table 1.** PICOS inclusion criteria for clinical trial eligibility.

PICOS^28^	Study inclusion criteria
Population	Post- and perimenopausal females (ages of 40–55) with psychosis or diagnosed with a primary psychotic disorder
Intervention	Antipsychotic treatment with an adjunct of either Estradiol or a SERM
Comparison	Antipsychotic treatment alone
Outcome	Clinical improvement measured with validated scales, primarily the Positive and Negative Symptom Scale
Study design	Interventional clinical trials (randomised or non-randomised), including pilot and feasibility studies, parallel-group and cross-over designs

Study eligibility criteria were defined by the authors and structured according to the PICOS framework.^28^

### Eligibility criteria

Eligible studies included post- and perimenopausal females aged 40+ diagnosed with a primary psychotic disorder, including those experiencing psychotic symptoms without a formal diagnosis. All primary psychotic disorders, as defined in the ICD-11, were included. Age cut-offs were determined based on the World Health Organization’s statistics on menopause.^30^

Studies were required to have the intervention of oestrogen treatment augmented with an antipsychotic. Oestrogen treatments included oestrogen hormone therapy (HT) of any kind or the use of SERMs. Interventions using oestrogen-progesterone combinations were also deemed appropriate. Studies that included oral and transdermal oestrogen were considered, as they have similar efficacy.^31^ There were no specifications for which antipsychotic was used in any dose.

Eligible designs were interventional clinical trials, including randomised or non-randomised pilot, feasibility, and parallel or cross-over trials. Studies that comprised populations outside post- and perimenopausal females were included, provided that the results for the target group were separated. Eligibility criteria are summarised in Table 1.

### Data synthesis and analysis

In August 2022, an electronic search was conducted with no language or date limitations on PubMed, EMBASE and PsycInfo. Searches combined terms related to psychotic disorders, oestrogen and selective oestrogen receptor modulators, and menopausal status. On PubMed, studies were filtered to ‘clinical trials’. On EMBASE and PsycInfo, there were no filters applied. EMBASE produced articles written in languages outside of English (*n* = 4), which were primarily Spanish. The titles and abstracts of these articles were translated, but none of them met the criteria for further review. Grey literature was searched using OPENGREY.EU – Grey Literature Database, yielding no relevant results. An additional search was completed on ClinicalTrials.gov to ensure that all clinical trials registered that were included. Study data were extracted and managed using a Microsoft Excel Spreadsheet that was automated to sort for duplicates. Titles and abstracts were screened for eligibility based on predefined criteria. Full texts were then assessed for inclusion. The full electronic search strategy is provided in the Supplemental Material.

Risk of bias assessment was conducted for randomised controlled trials using the Cochrane Risk of Bias Tool (ROB).^32^ The ROB results from each domain were created using an algorithm based on answers to the signalling questions. Studies were allocated a risk category based on the Cochrane Collaboration Handbook.^33^ Studies outside of the randomised controlled trials were assessed using the Joanna Briggs Institute (JBI) critical appraisal tools.^34^ JBI software ‘JBISUMARI’ was used to synthesise and guide the critical appraisal process. This software allowed for the merging of studies that have resulted from the same clinical trial without sacrificing data from each individual study, and the ability to use both the Cochrane Risk of Bias tool and JBI checklists in the same interface. A meta-analysis was not performed because of substantial heterogeneity in study design and outcome reporting, including incomplete and inconsistently reported PANSS data across trials.

## Results

### Study selection

**Figure 1.**
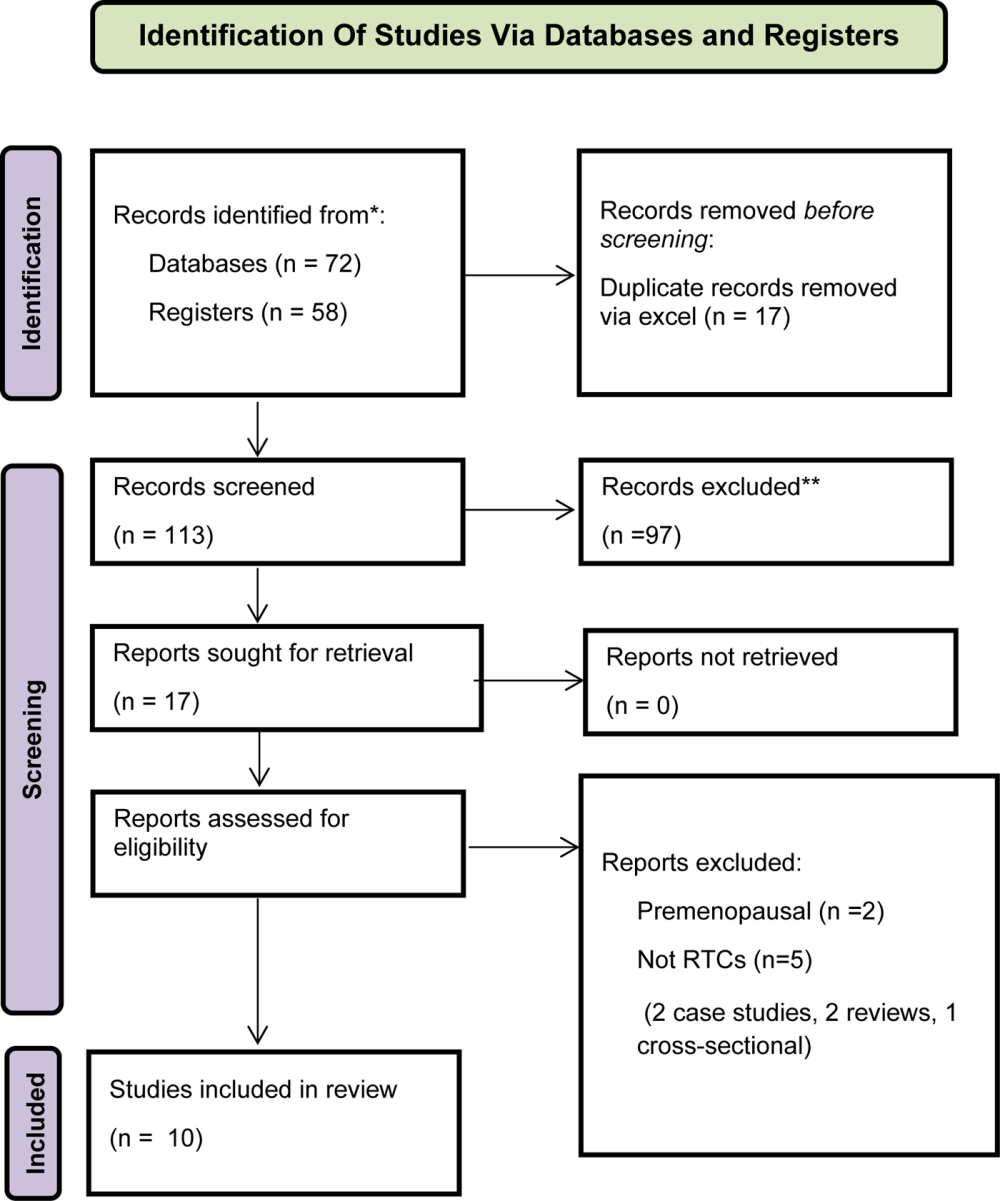
PRISMA^28^ flow diagram depicting the flow of information through the phases of a systematic review. PRISMA, Preferred Reporting Items for Systematic Reviews and Meta-Analyses.

Search results yielded: PubMed (*n* = 24), EMBASE (*n* = 28), PsycINFO (*n* = 20), and ClinicalTrials.gov (*n* = 58). Of the initial 130 articles identified, 17 were duplicates, and a total of 113 were screened. Title and abstract screening eliminated 96, as they did not meet the inclusion criteria. Ultimately, 17 full articles were reviewed and critically appraised and seven were eliminated. Two of these eliminated articles included females of childbearing age or premenopausal exclusively. The other five articles were excluded due to study type (two case reports, two trial reviews, one cross-sectional study). The study selection process is illustrated in Figure 1.

### Study characteristics

A total of 10 articles were extracted for this review (Table 2), representing nine unique clinical trials and involving 535 women receiving either an oestrogen agent (*n* = 290) or a placebo (*n* = 245). Oestrogen hormone therapy was used for the treatment of 63 women, while the SERM raloxifene was used to treat 227 women. Raloxifene doses ranged from 60 to 120 mg/day across trials, with treatment durations between 8 and 24 weeks; oestrogen-based interventions varied in formulation and dosing.

**Table 2.** Demographics of included studies.

Study	Design	Country	Setting	Participant characteristics	Agent	Outcomes measured	Description of main results
Bergemann 2005^13^	Double-blind placebo-controlled randomised cross-over study	Heidelberg, Germany	‘multicenter’	39 women; schizophrenia for the first time or relapse in psychotic symptoms that had sufficiently remitted after the acute phase. Results were split into groups based on age. <45 Receiving Estradiol/Placebo *n* = 29>45 Receiving Estradiol/Placebo *n *= 10	17β-estradiol + norethisterone acetate (dose dependent on menstrual stage)	Primary outcome: PANSS, overall condition was assessed using an 100-mm-Analogue-Scale Secondary outcome: BPRS, CGI, BDI	No difference vs placebo; estradiol augmentation did not improve relapse or psychopathology outcomes. Δ PANSS (treatment vs placebo): No significant change 95% CI: not provided p-Value: Not provided (reported as non-significant)
Kulkarni 2016^41^	Double-blind, placebo-controlled, randomised clinical trial	Melbourne, Australia	Inpatient and outpatient settings in two treatment centres, Alfred Health and Barwon Health	56 women, 40–70 yo; schizophrenia or schizoaffective disorder; perimenopausal and post-menopausal Raloxifene *n* = 26 Placebo *n *= 30	Raloxifene 120 mg/d	Primary outcome: PANSS Secondary outcome: MADRS, RBANS, Blood samples	Raloxifene produced a significantly greater reduction in PANSS relative to placebo Δ PANSS (treatment vs placebo): −6.37 95% CI: −11.64 to −1.10 p-Value: 0.02
Weiser et 2017^42^	Double-blind, placebo-controlled, randomised clinical trial	Romania and the Republic of Moldova	38 inpatient and outpatient sites in Romania and the Republic of Moldova between January 2011 and December 2012	200 women post-menopausal 45–65 yo; schizophrenia or schizoaffective Raloxifene *n* = 100 Placebo *n *= 100	Raloxifene 120 mg/d	Primary outcome: PANSS Secondary outcome: CGI-S, BACS, Simpson Angus Scale, UKU	Favours placebo; PANSS total significantly improved vs raloxifene Δ PANSS (treatment vs placebo): +4.5 (favours placebo) 95% CI: 2.3 to 6.7 p-Value: <0.001
Kianimehr 2014^43^	Double-blind, placebo-controlled, randomised clinical trial	Iran	Two universities affiliated psychiatric hospitals in Iran (Roozbeh and Razi Hospital)	50 post-menopausal women; schizophrenia Risperidone/Raloxifene *n*=25 Risperidone/Placebo *n* = 25	Raloxifene 120 mg/d	Primary outcome: PANSS	Improved positive symptoms only; no effect on negative or general symptoms Δ PANSS (treatment vs placebo): −14.2 (favours raloxifene) 95% CI: Not reported P-value: 0.18
Kulkarni 2010^40^	Double-blind Registered controlled trial Pilot Study	Australia	Not reported	35 (pooled) post-menopausal women who are 45 years or older; schizophrenia, schizoaffective disorder or schizophreniform disorder and currently unwell. Raloxifene 120 mg *n* = 13 Placebo *n* = 13 Raloxifene 60 mg* n = 9	Raloxifene 120* *mg/d Raloxifene 60* *mg/d	Primary outcome: PANSS Secondary outcome: MADRS	Dose-dependent effect; 120 mg raloxifene improved PANSS vs placebo, while 60 mg showed no effect. Δ PANSS (treatment vs placebo): Not reported (interaction favours treatment) 95% CI: Not reported p-Value: 0.01
Usall 2016^35^	Double-blind, placebo-controlled randomised clinical trial	Spain	Recruited from the inpatient and outpatient departments of Parc Sanitari Sant Joan de Déu, Hospital Universitari Institut Pere Mata, and Corporació Sanitària Parc Taulí	70 women; schizophrenia; post-menopausal status, receiving stable doses of their current antipsychotic medication Raloxifene *n* = 38 Placebo *n* = 32	Raloxifene 60* *mg/d	Primary outcome: PANSS Secondary outcome: SANS and Simpson Angus Rating Scale	Significant reduction in negative and total symptoms vs placebo. Δ PANSS (treatment vs placebo): −10.18 (favours raloxifene) 95% CI: Not directly reported for between-group Δ p-Value: 0.005
Labad 2016^36^*						Primary outcome: Genetic analysis obtained from peripheral blood samples combined with PANSS	Genetic variants in UGT1A8 and ESR1 genes modulate the treatment response to adding raloxifene to antipsychotic treatment in post-menopausal women with schizophrenia
Huerta-Ramos 2020^37^*						Primary outcomes: TAVEC, CPT II, TMT A and B, Stroop test, WAIS II, FAS	No significant difference in cognitive function
Usall 2011^38^	Double-blind, placebo-controlled, randomised clinical trial	Spain	Inpatient and outpatient departments of Parc Sanitari Sant Joan de Déu, Barcelona, Spain, and Corporació Sanitària Parc Taulí, Sabadell, Spain	33 women with schizophrenia, post-menopausal, have been receiving stable doses of their current antipsychotic medication Raloxifene *n* = 16 Placebo *n* = 17	Raloxifene 60 mg/d	Primary outcome: PANSS	Significant improvement across all PANSS subscales vs placebo Δ PANSS (treatment vs placebo): −7.47 (favours raloxifene) 95% CI: Not reported p-Value: 0.009
Huerta-Ramos 2014^39^*						Primary outcomes: UKU, TAVEC, CPT II, TMT, Stroop test, WAIS III, Phonetic fluency test (FAS) Secondary outcomes: PANSS PANSS, Simpson Angus Scale,	Significant differences in cognitive function specifically in some aspects of memory and executive function Memory (learning curve): *p* = 0.041 Executive function (FAS): *p* = 0.011

BACS, brief assessment of cognition in schizophrenia; BDI, beck depression inventory; BPRS, brief psychiatric rating scale; CGI, clinical global impression scale; CPT II, conner’s continuous performance test II; MADRS, montgomery-asberg depresssion rating scale; RBANS, repeatable battery for the assessment of neuropsychological status; SANS, scale for the assessment of negative symptoms; TAVEC, test de aprendizaje verbal españa–complutense; TMT, trail making test; UKU, udvalg for kliniske undersøgelser side effect rating scale; WAIS, wechsler adult intelligence scale.

Usall et al.^35^/Labad,^36^/Huerta-Ramos et al.^37^ and Usall et al.^38^/Huerta-Ramos^39^ reported results from the same trials and were merged accordingly, with primary sources designated for extraction. One article^40^ reported two separate pilot studies, which were analysed individually.

Of the nine trials, eight were randomised, placebo-controlled studies; one study used a cross-over design. Study durations ranged from 8 to 24 weeks. Trials were conducted across Australia, Spain, Germany, Iran, the United States, Romania, and Moldova.

Six trials included only post-menopausal participants, while two also included perimenopausal women. One trial included women of all ages but reported separate results for post-menopausal participants. In total, 506 participants met this review’s target population criteria, with a weighted mean age of 57.4 years.

### Risk of bias assessment

**Figure 2.**
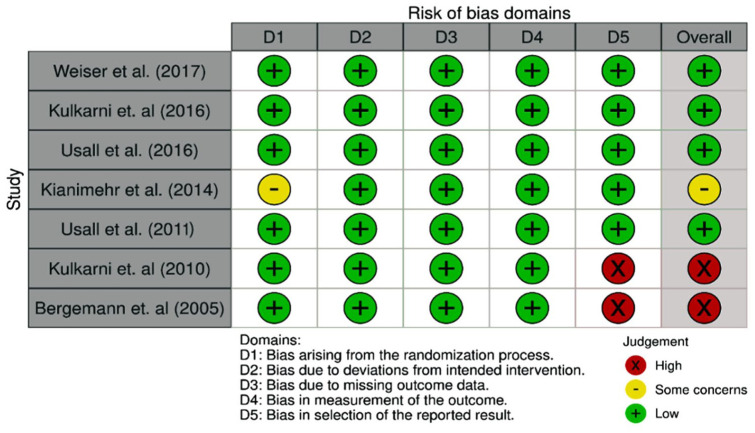
ROB traffic lights.^32^ Risk of bias was assessed using Cochrane Risk of Bias tool.^32^ ROB, risk of bias tool.

All randomised controlled trials were assessed using the Cochrane risk of bias 2.0 tool^32^ (see Supplemental Materials). Risk of bias assessments are summarised in Figure 2.

‘High risk’ was assigned to two studies, Kulkarni et al.^40^ and Bergemann et al.^13^ Kulkarni et al.’s^40^ pooled data from an unpublished pilot using raloxifene 60 mg/d, increasing the sample size and potentially overstating statistical power. Bergemann et al.^13^ inconsistently separated and combined data from women over and under 45 years and PANSS results were incompletely reported, with non-significant findings only briefly mentioned.

### Quality appraisal

All studies were appraised using the appropriate Joanna Briggs Institute checklist (see Supplemental Materials). Overall, no major concerns were found with the study methodology. However, several limitations may affect the interpretability and comparability of results.

In terms of participant selection, Kulkarni et al.^41^ screened 251 women but only enrolled 56. While exclusion reasons were partially listed, 106 were categorised as ‘other reasons’ without clarification, suggesting potential selection bias. Similarly, there were flaws in the methodology guiding Weiser et al.’s^42^ selection criteria, which lacked clear thresholds for illness severity and potentially contributed to their notably high baseline PANSS scores (mean = 101.5). Usall et al.^35^ also reported two screening errors: one participant menstruated during the study, and another had an unreported medication change. These errors were acknowledged but not fully explained, nor was the impact on trial outcomes investigated.

As previously mentioned, selective reporting further limits interpretation. Kulkarni et al.^40^ inflated the sample size and created greater statistical power for their results. Bergemann et al.^13^ report on two populations (women over 45 and women under 45) but pooled and separated them inconsistently. Since menopausal status was determined retrospectively, post-menopausal women only represented 26% of their sample and PANSS data for non-significant results were missing. In both Bergemann et al.^13^ and Kulkarni et al.,^41^ perimenopausal and postmenopausal participants were pooled, limiting the interpretation of hormonal influences.

Treatment design varies between studies. Kianimehr et al.^43^ initiated both raloxifene and a fixed dose of Risperidone 2 mg three times daily. Uniform dosing does not account for the heterogeneity of psychotic disorders and the patient's individual needs. Participants abstained from antipsychotics for only 1 week prior, which may not be an adequate washout period for drugs with long half-lives (e.g. aripiprazole), increasing the risk of carry-over effects. Initiating both drugs simultaneously also raises concerns about confounding, as improvements could stem from Risperidone alone. Bergemann et al.^13^ also risked carry-over effects, sequencing an A-B-A-B and B-A-B-A crossover design where the placebo and oestrogen HT groups switched during the trial. When medications are switched, there is a risk that the patients could potentially become ‘unblinded’ to their test group from either withdrawal or an adverse reaction following a new medication start.^44^

Thresholds for treatment adherence differed substantially between studies. Usall et al.^38^ accepted an 80% adherence, potentially allowing 16 missed doses and used pill counts to monitor. In contrast, Kulkarni et al.^41^ eliminated participants if they missed more than two doses of medication in the 12-week clinical trial period, creating a much stricter threshold.

Huerta-Ramos^37,39^ also had flaws in the results of output testing. Cognition was assessed using six distinct tests, all measuring different aspects of cognitive performance. Since this was an exploratory study, no parameters were pre-established as to what combination of tests or scores would define a change in cognition. Consequently, reliability and clinical significance are difficult to gauge.

### Synthesis of results

PANSS outcomes were mixed. Kulkarni et al.,^40,41^ Usall et al.,^35,38^ Kianimehr et al.,^43^ all reported statistically significant improvements with oestrogen-modulating agents. Usall et al.^38^ was the only one that reported solely a significant difference in positive symptom scores. Usall et al.^38^ observed significance only for positive symptoms, but their larger follow-up Usall et al.^35^ showed improvement in negative symptoms, leading the authors to suggest the initial result might have been a false positive. Improvements in negative symptoms were also reported in Kulkarni et al.^41^

Huerta-Ramos et al.^39^ also had conflicting results in their follow-up study. Their initial 2014 study indicated statistically significant improvements in the TAVEC learning curve (*p* = 0.041) and FAS phonetic fluency task (*p* = 0.011). Yet, they were unable to replicate these findings in their 2019 follow-up study, which was retrospectively thought to be attributed to the lower baseline PANSS scores.

Weiser et al.^42^ produced a paradoxical outcome where the placebo performed better than the raloxifene group. They theorised that this was potentially due to confounding from severe baseline illness (mean PANSS = 101.5), poor adherence, and/or site variability. The researchers also considered that this could be a legitimate finding and the possibility that raloxifene only works in stable outpatients and not severely decompensated individuals.

Similarly, Bergemann et al.^13^ reported no improvement in psychotic symptoms despite increased oestradiol levels. The authors noted that expected endocrine changes were observed, including lower prolactin and testosterone among clozapine-treated women, indicating that hormonal modulation occurred as anticipated without translating into symptomatic benefit. However, short-term symptomatic outcomes may be insufficient to capture more subtle effects on treatment responsiveness. Observational studies involving oestrogen HT have reported lower mean daily antipsychotic doses among women prescribed HRT,^45^ supporting the possibility that oestrogen exposure may enhance antipsychotic response and permit effective symptom control at lower doses over longer periods of time.^45^

Labad et al.^36^ found genomic variants associated with the participant’s response to raloxifene. From the 65 women that participated, four specific Taqman SNP genotyping assays corresponding to rs9340799, rs2234693 and rs1801132 in the ESR1 gene and rs1042597 in the UGT1A8 gene were genotyped. It was found that the rs2234693 variant in the ESR1 gene was correlated with poorer treatment response in terms of general psychopathology. These researchers also found that the rs1042597 variant in the UGT1A8 gene was linked to a distinct treatment response in negative symptoms with treatment using raloxifene. Since the rs1042597 linked to negative symptoms treatment response is a functional genetic variant, inconsistency in previous studies might be explained by the different expressions of this gene in the targeted population.

Across the included studies, most participants appeared to have established diagnoses of schizophrenia or related psychotic disorders, as inferred from inclusion criteria requiring prior diagnosis or ongoing antipsychotic treatment. However, none of the studies explicitly reported whether participants had new onset versus chronic psychosis during the menopausal transition. This ambiguity limits the interpretation of whether treatment effects differ by illness duration, and future trials should clearly stratify by illness stage.

For all studies, the overall dropout rates ranged from 15% to 26%, with most studies using intention-to-treat analyses to account for attrition. Due to the variations in outcomes and the polarising trend of research favouring raloxifene, there was consideration for the possibility of publication bias. The GRADE^46^ scoring tool was implemented (Table 3) to evaluate the credibility on the basis of RoB^32^ score, imprecision, inconsistency, indirectness, and publication bias.

**Table 3.** A summary of statistically significant evidence based on the PANSS domains.

Study	PANSS negative domain	PANSS positive domain	PANSS general symptoms domain	Total PANSS	Grade
Kulkarni 2016^41^	✘	✘	✔	✔	⨁⨁⨁◯ Moderate*
Usall 2016^35^	✔	✘	✔	✔	⨁⨁⨁⨁ High
Usall 2011^38^	✔	✔	✔	✔	⨁⨁⨁⨁ High
Bergemann 2005^13^	✘	✘	✘	✘	⨁⨁⨁◯ Moderate
Weiser et 2017^42^	✘	✘	✘	✘	⨁⨁⨁◯ Moderate
Kulkarni 2010^40^ (120 mg)	✘	✔	✔	✔	⨁◯◯◯ Very low*
Kulkarni 2010^40^ (60 mg)	✘	✔	✘	✘	⨁◯◯◯ Very low*
Kianimehr 2014^43^	✘	✔	✘	✘	⨁⨁⨁⨁ High

Source: GRADE.^46^

GRADE scale is used to assess the quality of evidence. High ⨁⨁⨁⨁: The author(s) have a lot of confidence that the true effect is similar to the estimated effect. Moderate ⨁⨁⨁◯: The author(s) believe that the true effect is probably close to the estimated effect. Low ⨁⨁◯◯: The true effect might be markedly different from the estimated effect. Very low ⨁◯◯◯: The true effect is probably markedly different from the estimated effect.

* Funding/associated with pharmaceutical companies.

✔, statistically significant findings; ✘, no statistical significance in the finding.

Three studies Usall et al.,^35,38^ Kianimehr et al.^43^ received the highest GRADE score. Weiser et al.^42^ was given a moderate score because of the inconsistent findings. Bergemann et al.^13^ was given a moderate score due to the missing data. The remaining studies all received lower scores because of a strong suspicion of publication bias.

## Discussion

Analysis of data identified consistent but modest evidence supporting the adjunctive use of raloxifene in improving psychotic symptoms, as reflected by significant reductions in total PANSS scores. Evaluation of the individual studies revealed that oestrogen HT had a statistically significant effect on negative symptoms of psychosis. Though given the small population size and study variability, evidence for traditional oestrogen HT remains inconclusive.

The small-to-moderate effect size associated with raloxifene underscores the potential clinical relevance of oestrogen pathways in schizophrenia pathophysiology, particularly during menopausal transition periods. These findings align with established hypotheses suggesting that oestrogen provides neuroprotection by modulating dopaminergic, serotonergic, and glutamatergic neurotransmission pathways, all critical targets implicated in schizophrenia.^47^ The potential for dopaminergic overactivity secondary to hypoestrogenism^48^ and antipsychotic-induced hyperprolactinemia^20^ further underscores oestrogen’s role in psychiatric symptomatology, highlighting a critical window for targeted therapeutic interventions during menopause.

The observed clinical effects of raloxifene may reflect receptor-specific mechanisms rather than global oestrogenic activity. Raloxifene exhibits selective agonist activity at central oestrogen receptors while acting as an antagonist in peripheral tissues,^49^ a profile that may preferentially engage ERβ- and GPER1-mediated pathways implicated in cortical and limbic regulation of psychosis-related circuits.^50^ This receptor bias demonstrated by raloxifene may help explain symptomatic benefits. Importantly, these mechanisms align with the observed heterogeneity in treatment response and suggest that oestrogenic modulation in psychosis is likely circuit- and receptor-dependent rather than driven by systemic hormone replacement alone.

Pharmacogenetic factors emerged as potential moderators of treatment response, notably genetic polymorphisms in oestrogen receptor (ESR1) and metabolising enzyme genes (e.g. UGT1A8). Labad et al.^36^ suggest that the CC genotype in rs2234693 is associated with poorer treatment outcomes in general psychopathology. Interestingly, this specific genotype has been related in the past to the risk of schizophrenia, specifically in terms of age of onset, indicating that females who display variations in the rs2234693 genotype display earlier onset of schizophrenia.^51^ Translationally, these findings suggest that genetic variation in ESR1 and UGT1A8 may contribute to treatment response heterogeneity.^52^ This supports pharmacogenetic stratification in future raloxifene trials, highlighting the need for a personalised medicine approach to managing psychosis in menopausal women. Therefore, incorporating hormonal status and pharmacogenetic stratification into future studies may make it easier to identify who benefits from raloxifene, rather than the effect being diluted in the overall average. Further pharmacogenetic research could facilitate individualised treatments, maximising therapeutic efficacy while mitigating adverse effects.

Effects on negative and cognitive symptoms varied. This variability may result from methodological inconsistencies, including the use of heterogeneous cognitive assessment tools and varying trial durations, suggesting the need for standardised, longitudinal evaluations. With this being said, a reduction in negative psychotic symptoms was replicated across three trials following the initiation of an oestrogen-modulating agent.^35,38,41^ Since negative symptoms respond poorly to antipsychotics^53^ there is a significant therapeutic gap which oestrogen modulators are promising candidates for. Despite cognitive symptoms significantly influencing functional outcomes,^54^ only a minority of studies explicitly examined cognitive performance, limiting the conclusions regarding oestrogen’s cognitive benefits. For this reason, future trials should prioritise cognitive and functional measures to address this significant gap.

Publication bias appeared to favour positive raloxifene findings, including those derived from studies with a higher risk of bias, particularly smaller pilot studies such as those reported by Kulkarni et al. This concern was further supported by conflicts of interest in several SERM studies and selective reporting signals, including pooled unpublished data and incomplete reporting of non-significant outcomes. Long-term trials with adequate power and methodological rigour remain necessary before oestrogen augmentation strategies can be formally recommended in clinical practice.

### Limitations

Limitations that should be taken into consideration are the small number of eligible trials with heterogeneous samples (oestrogen HT vs raloxifene), incomplete data, and probable publication bias. Follow-up durations were short, meaning there is limited evidence on long-term safety and adverse outcomes relevant to menopausal hormone-based treatments. Baseline hormonal status was inconsistently measured or reported across studies, limiting interpretation of whether treatment effects vary by degree of hypoestrogenism or by menopausal stage. Single reviewer screening adds further risk of error. AMSTAR rated the review moderate quality, owing to the lack of a second reviewer. The lack of prospective registration is acknowledged as a limitation.

## Conclusion

The current evidence supports further exploration of oestrogen modulation as adjunctive therapy in psychosis treatment during post- and perimenopause. Oestrogen-modulating agents have the potential to not only alleviate psychotic symptoms but also improve negative and cognitive symptoms that greatly affect quality of life and remain inadequately treated by antipsychotics alone. Across the available trial literature, raloxifene demonstrates the most consistent evidence of improvement, particularly for negative symptoms, although findings remain inconsistent and are partly informed by studies with a higher risk of bias. Given the heightened vulnerability associated with oestrogen deficiency during menopause, there is a critical clinical need for careful monitoring and tailored management strategies in this population. Further high-quality, adequately powered clinical trials incorporating comprehensive cognitive and functional assessments and exploring pharmacogenetic profiles are required to definitively make clinical recommendations and are essential to clarify therapeutic potential.

## Supplemental Material

sj-odt-1-tpp-10.1177_20451253261460148 – Supplemental material for Oestrogen modulators as augmentation to antipsychotics for the treatment of post- and perimenopausal psychosis: a systematic review Supplemental material, sj-odt-1-tpp-10.1177_20451253261460148 for Oestrogen modulators as augmentation to antipsychotics for the treatment of post- and perimenopausal psychosis: a systematic review by Cassidy Keen, Athanasios Hassoulas and Jill Richardson in Therapeutic Advances in Psychopharmacology

## Declarations

**Ethics approval and consent to participate:** Not applicable. This study is a systematic review and did not involve human participants or the collection of primary data.

**Consent for publication:** Not applicable.

**Availability of data and materials:** Not applicable.
