# Supplementary material for: Oestrogen modulators as augmentation to antipsychotics for the treatment of post- and perimenopausal psychosis: a systematic review
Source: Ther Adv Psychopharmacol. 2026 Jul 29;16:20451253261460148. doi: 10.1177/20451253261460148 (PMC13424932; doi:10.1177/20451253261460148)
Supplement: Supplementary file 1 [file sj-odt-1-tpp-10.1177_20451253261460148.odt]

Supplementary Material

Supplementary Table 1 

Quality analysis using Joanna Briggs Institute critical appraisal tools for Randomized Controlled Trials34

| Citation | Q1 | Q2 | Q3 | Q4 | Q5 | Q6 | Q7 | Q8 | Q9 | Q10 | Q11 | Q12 | Q13 |
| --- | --- | --- | --- | --- | --- | --- | --- | --- | --- | --- | --- | --- | --- |
| Kulkarni 2016 | Y | Y | Y | Y | Y | Y | Y | Y | Y | Y | Y | Y | Y |
| Usall 2015 | Y | Y | Y | Y | Y | Y | Y | Y | Y | Y | U | Y | Y |
| Usall 2011 | Y | Y | Y | Y | Y | Y | Y | Y | Y | Y | Y | Y | Y |
| Bergemann 2005 | Y | Y | Y | U | Y | Y | N | Y | Y | Y | Y | Y | Y |
| Weiser 2017 | U | Y | Y | Y | Y | Y | Y | Y | Y | Y | Y | Y | Y |
| Kulkarni 2010 | Y | Y | N | Y | U | Y | Y | N | Y | Y | N | N | Y |
| Kianimehr 2014 | U | Y | Y | Y | Y | Y | Y | N | Y | Y | Y | Y | Y |
| % | 71.42 | 100.0 | 85.71 | 85.71 | 85.71 | 100.0 | 85.71 | 71.42 | 100.0 | 100.0 | 71.42 | 85.71 | 100.0 |

Note. (Q1) Was true randomization used for assignment of participants to treatment groups? (Q2) Was allocation to treatment groups concealed? (Q3) Were treatment groups similar at the baseline? (Q4) Were participants blind to treatment assignment? (Q5) Were those delivering treatment blind to treatment assignment? (Q6) Were outcomes assessors blind to treatment assignment? (Q7) Were treatment groups treated identically other than the intervention of interest? (Q8) Was follow up complete and if not, were differences between groups in terms of their follow up adequately described and analyzed? (Q9) Were participants analyzed in the groups to which they were randomized? (Q10) Were outcomes measured in the same way for treatment groups? (Q11) Were outcomes measured in a reliable way? (Q12) Was appropriate statistical analysis used? (Q13) Was the trial design appropriate, and any deviations from the standard RCT design (individual randomization, parallel groups) accounted for in the conduct and analysis of the trial? Retrieved from Tufanaru et al. (2017)

Key: Y yes, N no, U unknown

Supplementary Table 2: JBI Checklist for Randomised Controlled Trials34

| Question | Description |
| --- | --- |
| Q1 | Was true randomisation used for assignment of participants to treatment groups? |
| Q2 | Was allocation to treatment groups concealed? |
| Q3 | Were treatment groups similar at the baseline? |
| Q4 | Were participants blind to treatment assignment? |
| Q5 | Were those delivering treatment blind to treatment assignment? |
| Q6 | Were outcomes assessors blind to treatment assignment? |
| Q7 | Were treatment groups treated identically other than the intervention of interest? |
| Q8 | Was follow-up complete and, if not, were differences between groups in terms of follow-up adequately described and analysed? |
| Q9 | Were participants analysed in the groups to which they were randomised? |
| Q10 | Were outcomes measured in the same way for treatment groups? |
| Q11 | Were outcomes measured in a reliable way? |
| Q12 | Was appropriate statistical analysis used? |
| Q13 | Was the trial design appropriate, and any deviations from the standard RCT design accounted for? |

| Supplementary Table 3: Cochrane ROB33 |
| --- |
| COChrane ROB | D1: Bias arising from the randomization process | D2: Bias due to deviations from intended intervention | D3: Bias due to missing outcome data | D4: Bias in measurement of the outcome | D5: Bias in the selection of the reported result | Overall |
| Weiser et al. (2017) | 1.1 Method of sequence not described. Being that this is a large trial run by an experienced trial it would not be unreasonable to indicate ‘probably yes’. Judgement: PY 1.2 Does not mention if the allocation sequence was concealed, due to the element of “quadruple blinding” ‘probably yes’ can be assumed. Judgement: PY 1.3 Demographics characteristics do not indicate bias in the randomisation process. “Demographics and clinical characteristics of the 200 participants are presented in Table 1, showing no detectable differences between the two groups” “P=.193” Judgement: N | 2.1 Its masking is quadruple (Participant, Care Provider, Investigator, Outcomes Assessor). Judgement: N 2.5 “using an unmodified intention-to-treat approach (last observation carried forward), a modified intention-to-treat analysis using all subjects who had at least 1 follow-up value, and a completers-only approach using data from all subjects with observed data at 16 weeks.” Judgement: Y | 3.1 All data accounted for “A total of 26 women did not complete the study, and rates of early termination in the placebo group (n=16) and raloxifene group (n=10) did not differ significantly between groups (P = .193) (Figure 1). Demographic and clinical characteristics of the 200 participants are presented in Table 1, showing no detectable differences between the 2 groups.” Judgement: Y | 4.1 Judgement: N 4.2 “Symptoms were assessed at baseline and at predetermined Intervals” Judgement: N | 5.1 Judgement: Y 5.2 Numerous scores reported Judgement: N 5.3 Judgement: N | Study is judged to be at low risk of bias for all domains |
| Kulkarni et al. (2016) | 1.1 “ The Alfred Clinical Trials  pharmacist will organise randomisation and has extensive experience with this procedure” Judgement: Y 1.2 “Randomisation Procedure: Upon entry into the double-blind phase, each patient will be allocated an identification number and randomly assigned to a treatment regimen according to a computer generated pseudo-random code. Equal numbers will be assigned to both treatment arms” Randomization was assigned by a “computer-generated 1:1 block randomization schedule” Judgement: Y 1.3 Demographics don’t appear differ significantly Age of randomisation (p= .95); age at onset, mean (p=.85); antipsychotic use, mean (p=.13); total antipsychotic dose in risperidone equivalents (p=.33); antidepressant use (.18), mood stabilizer use (p=.18), anxiolytic use (p= >.99), clozapine treatment (p= .45) Judgement: N | 2.1 2.2 “Patients, raters and clinicians are “blind” as to whether each patient receives adjunctive SERM or placebo.” Judgement: N 2.6 “Analyses were conducted using STATA software with the α value set at 05. Intention-to-treat analysis was conducted, and missing data imputations were not performed.” Judgement: Y | 3.1 Judgement: Y | 4.1 Judgement: N 4.2 Judgement: N 4.3 Judgement: N | 5.1 There were some results adjusted for type of diagnosis, age of onset, age at baseline, antipsychotic dosage at baseline and receiving clozapine at baseline Judgement: PY 5.2 Numerous scores reported Judgement: N 5.3 Judgement: N | Study is judged to be at low risk of bias for all domains |
| Usall et al. (2011) | 1.1 33 Subjects were randomised by the Parc Sanitari Sant Joan de Deu trial pharmacy. Does not say exact sequence used. Participants were blinded for this. Judgement: Y 1.2 P score shows no statistical differences in groups Judgement: N | 2.1 2.2 All study personnel and participants remained blinded to treatment assignment. Judgement N 2.6 Used PANSS rating scale Judgement: N | 3.1 One person in each group lost to the final assessment and the data from the last observation was used to carry over the results. Since the sample size was small losing one participant meant for the Raloxifene group losing final results for 6.25% of the population and for the control group losing 5.88% of the group Judgement: PN 3.2 Used the “last carried over method” Judgement: Y | 4.1 Judgement: N 4.2 Judgement: N 4.3 Judgement: N | 5.1 Judgement: PY 5.2 Numerous scores reported Judgement: N 5.3 Judgement: N | Study is judged to be at low risk of bias for all domains |
|  | D1: Bias arising from the randomization process | D2: Bias due to deviations from intended intervention | D3: Bias due to missing outcome data | D4: Bias in measurement of the outcome | D5: Bias in the selection of the reported result | Overall |
| Kianimehr et al. (2014) | 1.1 States Randomized but doesn’t detail the process of randomisation. Author does appear to have extensive experience or previous randomised controlled trials to fall back on. Judgement: NI 1.2 “Risperidone (6 mg/day in 3 divided doses) (Janssen Pharmaceuticals, Toronto, Canada; 2 mg tablet) combined with either placebo (N = 23) or 120 mg/day of raloxifene.” – Since they are getting it compounded specifically for the study, it would be reasonable to assume the authors took into consideration concealing the medications Judgement: PY 1.2 No significant differences in study population. Judgement: N | 2.1 2.2 Medication was administered under placebo-controlled, double-blind conditions Judgement: N Judgement: N 2.6 Judgement Y | 3.1 Judgement: Y | 4.1 Judgement: N 4.2 Judgement: N 4.3 Judgement: N | 5.1 Judgement: Y 5.2 Numerous scores reported Judgement: N 5.3 Judgement: N | Some Concerns: The study is judged to raise some concerns in at least one domain for this result. |
| Usall et al. (2015) | 1.1 “Once a patient accepted inclusion in the study and was assessed, the investigator called the pharmacy of the Parc Sanitari Sant Joan de Déu for group assignment. The pharmacy randomly assigned patients to one of the 2 treatment groups with a proportion of 1:1 (blocks of 4 patients, 2 for group 1 and 2 for group 2), based on a random number list.” Judgement: Y 1.2 “Medication was administered under placebo-controlled, double-blind conditions. Medication was prepared by the pharmacy of the participating hospital. Placebo tablets were prepared so as to be identical in appearance to the raloxifene tablets. The compounds from both treatment arms (raloxifene and lactose as placebo) were over-encapsulated in dark green gelatin capsules in order to assure blinding” Judgement: Y 1.3 At baseline there were significant differences between the groups in the general psychopathological PANSS subscale ( P =.020, the raloxifene group scoring higher than the placebo) Judgement: N | 2.1 2.2 Medication was administered under placebo-controlled, double-blind conditions Judgement: N Judgement N 2.6 “These patients were included in the analysis since it was carried out in accordance with intention to treat” Judgement Y | 3.1 “Only patients with at least baseline and 4 weeks’ follow-up data were included (70 patients in total, 32 placebo, 38 raloxifene), and last-observation-carried-forward method was applied to patients with missing data at 12 or 24 weeks’ follow-up. SPSS 19.0 (IBM Corp.) was used for statistical analysis; P -values <.05 were considered significant.” Judgement: PN 3.2 Used the “last carried over method” Judgement: Y | 4.1 Evaluations were performed by 3 psychologists, who previously performed inter-rater reliability tests. Each psychologist evaluated patients from only 1 center. Psychopathological symptoms were assessed at baseline and at weeks 4, 12, and 24 using the PANSS. 26 Negative symptoms were also assessed with the Scale for the Assessment of Negative Symptoms (SANS). Twenty nine side effects were assessed at each visit, and the Simpson-Angus Rating Scale 30 was administered for extrapyramidal side effects as was the UKU side effect rating scale. Judgement: N 4.2 Judgement N 4.3 All study personnel and participants remained blind to treatment assignment for the duration of the study. Judgement N | 5.1 Judgement: PY 5.2 Numerous scores reported Judgement: N 5.3 Judgement: N | Study is judged to be at low risk of bias for all domains |
|  | D1: Bias arising from the randomization process | D2: Bias due to deviations from intended intervention | D3: Bias due to missing outcome data | D4: Bias in measurement of the outcome | D5: Bias in the selection of the reported result | Overall |
| Kulkarni et al. (2010) | 1.1 1.2 “participants were randomized by the Alfred Clinical Trials Pharmacy to receive either daily oral raloxifene HCl (120 mg/day in the current trial; 60 mg/day in the previous trial) or oral placebo according to a block randomization schedule. Medications were dispensed in capsule form, at a dose of one capsule/day.” Judgement: Y Judgement: Y 1.3 “Data for 35 women were included in this study. Mean baseline demographic and clinical characteristics for participants in each group are presented in Table 1. Groups were matched on age, duration of illness, and positive, general and total PANSS scores.” The 60 mg/day raloxifene HCl group had significantly lower baseline negative PANSS, MADRS and SAS ratings. Judgement: PY | 2.1 2.2 All study personnel and participants remained blinded to treatment assignment. Judgement N 2.6 “Psychopathology was assessed at baseline and weeks 2, 4, 6, 8, 10 and 12 using the PANSS, a 30-item clinician-rated measure of positive, negative and general symptoms of schizophrenia. Depressive symptoms were also assessed fortnightly using the 10-item clinician-rated Montgomery-Åsperg Depression Rating Scale (MADRS).” Judgement: N | 3.1 All data available but limited sample Judgement: Y | 4.1 Judgement: N 4.2 Judgement: N 4.3 Judgement: N | 5.1 Data results were pooled from a very small population (n=33). Pooled results (n=9) were from a previous “unpublished” study that the researchers were aware of the results of prior to this study taking place. Not blinded to the outcomes due to pooled data. Judgement: N 5.2 Using numerous scales Judgement: N 5.3 using a data set from a pervious trial to demonstrate the performance of the current trial allows for selective reporting Judgement: Y | High risk of bias: The study has been at high risk of bias in at least one domain |
| Bergemann et al. (2005) | 1.1 1.2 “multicenter, randomized, placebo-controlled, double-blind, cross-over study based on an A–B–A–B (and/or B–A–B–A) design was applied” Judgement: Y Judgement: Y 1.3 Some differences in population, also had large inclusion criteria. The 10 women above the age of 45 does not seem to be representative of that population (n=29 women over the age of 45) Judgement: PN | 2.1 2.2 All study personnel and participants remained blinded to treatment assignment. Judgement N 2.6 “The target parameter of the investigation was the improved therapeutic effectiveness of a combination of antipsychotics and estradiol versus an antipsychotic monotherapy for relapse prevention. To assess the state of psychopathology, the German versions or translations of the following standard rating scores and/or self-rating scales were applied: The Positive and Negative Syndrome Scale, Brief Psychiatric Rating Scale, Clinical Global Impressions, Beck Depression Inventory. The overall condition was assessed by using an 100-mm-Analogue-Scale.” Judgement: N | 3.1 Judgement: Y | 4.1 Judgement: N 4.2 Judgement: N 4.3 Judgement: N | 5.1 Judgement: PY 5.2 5.3 Pools women both below and above the age of 45 for some results but not others. There is no table or any statistical data reported for PANSS Judgement Y | High risk of bias: The study has been at high risk of bias in at least one domain |

Supplementary File: Full Search Strategy

Search Date: August 2022
Language Limits: None
Date Limits: None (initially considered a 10-year limit, then removed)

Databases Searched:

- PubMed

- EMBASE

- PsycINFO

- ClinicalTrials.gov

- OpenGrey.eu

Filters Applied:

- PubMed: Clinical Trials

- EMBASE & PsycINFO: No filters

- ClinicalTrials.gov & OpenGrey: Manual review

Search String Used:

{(psychosis OR psychotic OR psychotic symptoms OR psychotic disorders OR schizophrenia OR delusional disorder OR schizoaffective disorder) AND (Oestrogen OR Estrogens OR Estrone OR Estradiol OR Estriol OR SERMs OR Selective Estrogen Receptor Modulators OR hormone therapy OR Raloxifene OR Tamoxifen OR Bazedoxifene) AND (menopause OR perimenopause OR post-menopause)}. 

Total Results Identified Before Screening:

- PubMed: 24

- EMBASE: 28

- PsycINFO: 20

- ClinicalTrials.gov: 58

- OpenGrey.eu: 0

Supplementary Checklist 1: PRISMA Checklist28

| Section and Topic | Item # | Checklist item | Location where item is reported |
| --- | --- | --- | --- |
| TITLE |  |
| Title | 1 | Identify the report as a systematic review. | Title page |
| ABSTRACT |  |
| Abstract | 2 | See the PRISMA 2020 for Abstracts checklist. | Abstract section |
| INTRODUCTION |  |
| Rationale | 3 | Describe the rationale for the review in the context of existing knowledge. | Introduction pg 3, 4 |
| Objectives | 4 | Provide an explicit statement of the objective(s) or question(s) the review addresses. | Introduction pg 4 |
| METHODS |  |
| Eligibility criteria | 5 | Specify the inclusion and exclusion criteria for the review and how studies were grouped for the syntheses. | Eligibility criteria pg 4, 5 |
| Information sources | 6 | Specify all databases, registers, websites, organisations, reference lists and other sources searched or consulted to identify studies. Specify the date when each source was last searched or consulted. | Data synthesis and analysis pg 5 |
| Search strategy | 7 | Present the full search strategies for all databases, registers and websites, including any filters and limits used. | Data synthesis and analysis pg 5 |
| Selection process | 8 | Specify the methods used to decide whether a study met the inclusion criteria of the review, including how many reviewers screened each record and each report retrieved, whether they worked independently, and if applicable, details of automation tools used in the process. | Data synthesis and analysis pg 5 |
| Data collection process | 9 | Specify the methods used to collect data from reports, including how many reviewers collected data from each report, whether they worked independently, any processes for obtaining or confirming data from study investigators, and if applicable, details of automation tools used in the process. | Search strategy pg 4 & Data synthesis and analysis pg 5 |
| Data items | 10a | List and define all outcomes for which data were sought. Specify whether all results that were compatible with each outcome domain in each study were sought (e.g. for all measures, time points, analyses), and if not, the methods used to decide which results to collect. | PICOS inclusion criteria pg 5 |
| 10b | List and define all other variables for which data were sought (e.g. participant and intervention characteristics, funding sources). Describe any assumptions made about any missing or unclear information. | PICOS inclusion criteria pg 5 |
| Study risk of bias assessment | 11 | Specify the methods used to assess risk of bias in the included studies, including details of the tool(s) used, how many reviewers assessed each study and whether they worked independently, and if applicable, details of automation tools used in the process. | Data synthesis and analysis pg 5 |
| Effect measures | 12 | Specify for each outcome the effect measure(s) (e.g. risk ratio, mean difference) used in the synthesis or presentation of results. | Study selection pg 6 |
| Synthesis methods | 13a | Describe the processes used to decide which studies were eligible for each synthesis (e.g. tabulating the study intervention characteristics and comparing against the planned groups for each synthesis (item #5)). | Data synthesis and analysis pg 5 |
| 13b | Describe any methods required to prepare the data for presentation or synthesis, such as handling of missing summary statistics, or data conversions. | Data synthesis and analysis pg 5 |
| 13c | Describe any methods used to tabulate or visually display results of individual studies and syntheses. | Data synthesis and analysis pg 5 |
| 13d | Describe any methods used to synthesize results and provide a rationale for the choice(s). If meta-analysis was performed, describe the model(s), method(s) to identify the presence and extent of statistical heterogeneity, and software package(s) used. | Data synthesis and analysis pg 5 |
| 13e | Describe any methods used to explore possible causes of heterogeneity among study results (e.g. subgroup analysis, meta-regression). | Data synthesis and analysis pg 5 and synthesis of results pg 9 |
| 13f | Describe any sensitivity analyses conducted to assess robustness of the synthesized results. | Synthesis of results pg 9 |
| Reporting bias assessment | 14 | Describe any methods used to assess risk of bias due to missing results in a synthesis (arising from reporting biases). | Synthesis of results pg 10 |
| Certainty assessment | 15 | Describe any methods used to assess certainty (or confidence) in the body of evidence for an outcome. | Synthesis of results pg 10 |
| RESULTS |  |
| Study selection | 16a | Describe the results of the search and selection process, from the number of records identified in the search to the number of studies included in the review, ideally using a flow diagram. | Figure 1 PISMA flow diagram |
| 16b | Cite studies that might appear to meet the inclusion criteria, but which were excluded, and explain why they were excluded. | Figure 1 PISMA flow diagram |
| Study characteristics | 17 | Cite each included study and present its characteristics. | Study characteristics pg 6 |
| Risk of bias in studies | 18 | Present assessments of risk of bias for each included study. | Risk of bias assessment pg 8 |
| Results of individual studies | 19 | For all outcomes, present, for each study: (a) summary statistics for each group (where appropriate) and (b) an effect estimate and its precision (e.g. confidence/credible interval), ideally using structured tables or plots. | Synthesis of results pg 9 |
| Results of syntheses | 20a | For each synthesis, briefly summarise the characteristics and risk of bias among contributing studies. | Synthesis of results pg 9 and 10 |
| 20b | Present results of all statistical syntheses conducted. If meta-analysis was done, present for each the summary estimate and its precision (e.g. confidence/credible interval) and measures of statistical heterogeneity. If comparing groups, describe the direction of the effect. | Synthesis of results pg 9 and 10 |
| 20c | Present results of all investigations of possible causes of heterogeneity among study results. | Synthesis of results pg 9 and 10 |
| 20d | Present results of all sensitivity analyses conducted to assess the robustness of the synthesized results. | Synthesis of results pg 9 and 10 |
| Reporting biases | 21 | Present assessments of risk of bias due to missing results (arising from reporting biases) for each synthesis assessed. | Synthesis of results pg 9 and 10 |
| Certainty of evidence | 22 | Present assessments of certainty (or confidence) in the body of evidence for each outcome assessed. | Synthesis of results pg 9 and 10 |
| DISCUSSION |  |
| Discussion | 23a | Provide a general interpretation of the results in the context of other evidence. | Discussion pg 11/12 |
| 23b | Discuss any limitations of the evidence included in the review. | Discussion pg 11/12 |
| 23c | Discuss any limitations of the review processes used. | Discussion pg 11/12 |
| 23d | Discuss implications of the results for practice, policy, and future research. | Discussion pg 11/12 & Conclusion 13 |
| OTHER INFORMATION |  |
| Registration and protocol | 24a | Provide registration information for the review, including register name and registration number, or state that the review was not registered. | Search strategy pg 4 |
| 24b | Indicate where the review protocol can be accessed, or state that a protocol was not prepared. | Data synthesis and analysis pg 5 and synthesis of results pg 9 |
| 24c | Describe and explain any amendments to information provided at registration or in the protocol. | Limitations pg 11 |
| Support | 25 | Describe sources of financial or non-financial support for the review, and the role of the funders or sponsors in the review. | Declarations pg 13 |
| Competing interests | 26 | Declare any competing interests of review authors. | Declarations pg 13 |
| Availability of data, code and other materials | 27 | Report which of the following are publicly available and where they can be found: template data collection forms; data extracted from included studies; data used for all analyses; analytic code; any other materials used in the review. | Declarations pg 13 |

From:  Page MJ, McKenzie JE, Bossuyt PM, Boutron I, Hoffmann TC, Mulrow CD, et al. The PRISMA 2020 statement: an updated guideline for reporting systematic reviews. BMJ 2021;372:n71. doi: 10.1136/bmj.n71. This work is licensed under CC BY 4.0. To view a copy of this license, visit [https://creativecommons.org/licenses/by/4.0/](https://creativecommons.org/licenses/by/4.0/)
